# Social Anxiety Disorder: Associated Conditions and Therapeutic Approaches

**DOI:** 10.7759/cureus.32687

**Published:** 2022-12-19

**Authors:** Nasser A Alomari, Sohaila K Bedaiwi, Abdulrahman M Ghasib, Ammar J Kabbarah, Saad A Alnefaie, Nada Hariri, Munirah A Altammar, Abdulaziz M Fadhel, Fai M Altowairqi

**Affiliations:** 1 Family Medicine, King Fahad Armed Forces Hospital, Jeddah, SAU; 2 Inpatient Pharmacy, King Abdullah Medical City, Makkah, SAU; 3 Emergency Department, Prince Meshari Bin Saud Hospital, Al-Baha, SAU; 4 College of Medicine, King Abdulaziz University, Jeddah, SAU; 5 Emergency Department, Alnoor Specialist Hospital, Makkah, SAU; 6 Department of Internal Medicine, Amiri Hospital, Kuwait City, KWT; 7 Intensive Care Unit Department, King Faisal Hospital, Makkah, SAU

**Keywords:** social phobia, panic disorder, obsessive compulsive disorder, mental health, behavioral therapy

## Abstract

Social anxiety disorder (SAD) is a highly distressing chronic psychiatric disorder characterized by persistent fear of social situations in anticipation of being judged negatively by others. As shyness mimics some of the symptoms of SAD, people suffering from this debilitating disease are often underdiagnosed. It can have a devastating impact on all areas of life including academic performance, social growth, relationship status, and work performance. In recent years, research on anxiety and related disorders has proliferated due to the increased use of cognitive-behavioral models. The understanding of SAD has evolved greatly from distinguishing it from shyness to implementing modalities to comprehend the sophisticated underlying mechanism of disease prevalence and progression as well as methods to treat it. This review summarizes the concept of SAD, its epidemiology, symptoms, and diagnostic tools. Frequent comorbidities including other psychiatric disorders are also discussed. Additionally, we examine the latest evidence related to treatment options including psychotherapy and pharmacotherapy as well as recommendations for managing SAD.

## Introduction and background

Social anxiety disorder (SAD) is a debilitating social phenomenon characterized by persistent fear of social situations due to anticipation of negative judgment by others [1]. The prevalence of SAD is estimated to be around 12% [2]. SAD must be differentiated from shyness because the latter does not cause serious mental disability or interfere with the majority of life events. SAD has a profound impact on the quality of life of the affected individual. Educational achievement can be undermined, with a heightened risk of leaving school early and resulting in poor academic performance and qualifications [3]. A survey reported that people with generalized SAD had 10% lower wages compared to the general population [4]. On average, individuals with SAD have fewer friends and have more difficulty getting along with friends [5]. They are less likely to marry and have children and more likely to divorce [6].

The Diagnostic and Statistical Manual of Mental Disorders, Fifth Edition (DSM-5) criteria for SAD include eight components including fear of social situations, continuous triggering of anxiety by social situations, exaggerated fear that is out of proportion to the seriousness of the threat posed by social situations, avoidance of social situations due to intense fear, impaired social or occupational functions, the persistence of fear for six months, the fear not being attributed to any substance abuse, symptoms that cannot be explained by another mental illness, and avoidance that is not related to a medical condition such as Parkinson's disease or obesity [7]. However, the impact of SAD can be mild, moderate, or extreme. Some people suffering from SAD can manifest symptoms in certain specific events like performing in front of an audience or eating around others, while others can experience symptoms in all forms of social interactions [8]. People who suffer from mild social anxiety might encounter physical and psychological symptoms of social anxiety but can participate in or endure social situations [9]. However, they may exhibit symptoms of SAD in specific social situations. A person with moderate SAD can participate in some social situations while avoiding other types of social situations such as public speaking or group discussions. People with extreme social anxiety may experience more severe symptoms of social anxiety, such as panic attacks in social situations. They often struggle to cope socially with daily life situations. Such social experiences often discourage people with severe SAD from having any social interactions [10]. 

Although normal shyness and mild SAD can exhibit similar symptoms on some occasions, they need to be distinguished from each other [11]. To be nervous or stressed in challenging situations such as public speaking in front of a crowd or giving a lecture can be regarded as shyness [12]. However, SAD involves persistent fear that prompts individuals to avoid a social event due to the fear of being judged negatively by others. The symptoms of social anxiety are more severe, including palpitation, tremulousness, blushing, and diaphoresis, which culminate in the loss of optimal functioning [13]. Dell’Osso et al. (2014) used DSM-5 for the dimensional assessment of anxiety symptoms among university students and their effect on functional impairment. They reported seven physical symptoms related to social situations among subjects who reported experiencing SAD physical symptoms during childhood/adolescence: (1) quivering, (2) embarrassment or feeling flushed, (3) heart pounding or palpitation, (4) feeling dizzy or about to faint, (5) sweating or diaphoresis, (6) diarrhea, nausea, or stomach ache, and (7) having the urge to urinate [14]. 

Different parameters have been proposed for self- or clinician-administered SAD assessment. The first clinician-administered scale of SAD was the Liebowitz Social Anxiety Scale (LSAS). The scale is divided into two subscales based on two measures, i.e., social interactions and performance situations [12]. Several self-report questionnaires are available to analyze the incidence of SAD. Social Phobia and Anxiety Inventory (SPAI) is a self-report inventory regarding the symptoms, cognition, and behavior screening in certain potential situations. SPAI is effective in distinguishing between panic disorders and agoraphobia, and SAD [15]. Some of the other rating scales have certain limitations, including a narrow focus on one aspect rather than providing a broader picture. To rectify this limitation, the Social Phobia Inventory (SPIN) was designed to comprehensively evaluate fear, avoidance, and physiological symptoms [16]. The current review is aimed at providing the latest evidence related to treatment options and recommendations for managing SAD.

## Review

Associated conditions

Anxiety disorder is one of the most prevalent types of mental disorders, representing about 18% of the total psychiatric disorder cases. Currently, the worldwide prevalence of anxiety disorders is estimated at 7.3% [17]. Among them, specific phobias constitute the highest proportion, at 10.3%, followed by panic disorder (PD), SAD, and generalized anxiety disorder (GAD). Women seem to be more prone to anxiety disorders compared to men, with females being 1.5 to 2 times more prone to having these disorders compared to males [18]. Often, anxiety disorders are accompanied by comorbid depressive disorders. For example, SAD exhibits confounding factors of comorbidity with PD, agoraphobia, atypical depression, and body dysmorphic disorder, which makes its differential diagnosis extremely challenging. SAD can be distinguished from PD because panic attacks in PD are recurrent, whereas, in SAD, panic attacks are associated with embarrassing social interactions. Agoraphobia can be differentiated from SAD based on the fact that agoraphobic individuals demonstrate symptoms of anxiety when escape seems impossible, such as in a shopping mall, whereas an individual suffering from SAD fears such a setting due to fear of social interactions. Individuals with body dysmorphic disorder can be distinguished from those with SAD as the former are excessively concerned about their appearances, irrespective of social occasions [19]. Although the prevalence of anxiety disorders is quite high, their clinical diagnosis remains low in primary care. The co-presentation of different disorders concurrently results in low treatment effectiveness. To evaluate a specific therapeutic intervention, an assessment of symptoms is important. The symptoms can also vary based on the patient's age. Recent investigations into diagnostic challenges in PD suggest that older people are less likely to exhibit pronounced panic symptoms and levels of anxiety compared to young adults [20]. However, some previous studies have contradicted these findings as they reported no significant difference between age groups in terms of symptomatology [21,22].

Panic Disorder (PD)

PD is characterized by recurrent panic attacks and is associated with a significant functional disability when complicated by agoraphobia [23]. The DSM defines PD as “an abrupt surge of intense fear or discomfort”. The common symptoms of PD are smothering, derealization, frenzy, paresthesias, and feelings of being choked [24]. The diagnosis of PD should be based on the updated diagnostic criteria of DSM-5. The upgraded version of DSM maintains a distinction between agoraphobia and PD by defining panic attacks as a specifier. In DSM-5, the operational definition of PD includes two overlapping criteria for the diagnostic evaluation. While criterion A focuses on the recurrence of panic attack-related conditions for over a month, criterion B involves concerns and behavioral anomalies exhibited as a consequence of PD [25]. A wide spectrum of panic attacks can be observed in PD, ranging from multiple attacks in an hour to only a few within a year. Although panic attacks are also reported in other conditions including anxiety, mood, and substance use disorders, the key feature of PD-related panic attacks is their abrupt onset [26]. A differential diagnosis of PD disorder is critical to distinguish it from other mental disorders. The anxiety symptoms reported by PD patients tend to be physical rather than psychological as seen in GAD and SAD. Selective serotonin reuptake inhibitors (SSRIs) and benzodiazepines are the standard options for the treatment of PD [27].

Generalized Anxiety Disorder (GAD)

Generalized anxiety disorder (GAD) is a chronic disorder characterized by persistent worry symptoms for at least six months [28]. GAD is the most commonly reported (22%) anxiety disorder in primary care in patients who present with symptoms of anxiety. The overall prevalence of GAD among the general public is 1.9-5.1% [29]. Clinical manifestations of GAD include excessive worrying, anxiety, and hypervigilance. The age of onset of GAD differs from other psychiatric disorders. The detailed diagnostic evaluation criteria for GAD are mentioned in DSM-5. According to DSM-5, a diagnosis of GAD should be made based on the following aspects: excessive anxiety symptoms occurring on more days than not for at least six months; inability to control worries; the manifestation of at least three out of six symptoms including restlessness, being easily fatigued, inability to concentrate, irritability, muscle tension, and sleep disturbances; impairment of social and occupational life; and GAD disturbance not due to the physiological effects of any substance [30]. GAD should be differentiated from other anxiety disorders as emotional intensity and fear of expression vary greatly among mental disorders. Turk et al., in their study, concluded that individuals experiencing GAD expressed greater emotional intensity compared to people with SAD and the control population. Their investigations also revealed that individuals with SAD were less perceptive of their emotions and had more difficulty in expressing them compared to people with GAD and the control group [31]. The prevalence rates of GAD increase with age. The age-related dynamic of GAD was analyzed by Diefenbach et al., who reported that health-related worries were predominant among older adults compared to younger adults [32]. These findings signify the differences in the qualitative nature of GAD among different age groups. Females are at increased risk of developing GAD compared to men, with a significant correlation to chronic medical illnesses. GAD comorbidity with depression is a common clinical feature [33].

Obsessive-Compulsive Disorder (OCD)

OCD is a chronic condition and a significant cause of mental disability globally. OCD is now categorized under "obsessive-compulsive and related disorders", as a group of diseases by DSM-5 [34]. The other conditions grouped with OCD include body-dysmorphic disorder (BDD), hoarding disorder, excoriation (skin-picking) disorder, and unspecified obsessive-compulsive and related disorders. OCD is a condition that involves intrusive thoughts that provoke a level of discomfort [35]. To reduce the distress associated with OCD, the patients exhibit some form of excessive compulsion to satisfy their obsessive feelings. The commonly reported obsessions among OCD patients include fear of contamination, sexual fears, and fear of aggression. To overcome these thoughts, the patients engage in compulsive behaviors that can include aggressive cleaning, reassurance-seeking, and repeating things [36]. In the DSM-5, several modifications have been incorporated regarding OCD definitions and diagnostic measures. Apart from a change in classification, OCD is now characterized by the presence of obsessions, compulsions, or both. The new definition of obsessions is based on two parameters instead of four as in DSM-4. The first parameter includes recurrent thoughts and urges, which, if experienced, cause marked anxiety and distress. The second parameter involves the individual's attempts to suppress such thoughts through the performance of compulsive actions. The diagnostic identification of compulsions includes repetitive behaviors and mental acts such as praying, counting, or repeating words over and over in response to satisfying an obsession. Behavioral acts are usually an attempt to prevent distress. According to DSM-5, behaviors associated with OCD are time-consuming and often result in patients wasting away almost one hour per day on obsessions and compulsions, which leads to social and occupational impairments. The disturbance is not caused by any physiological factor [30]. The differential diagnosis of OCD from other mental disorders such as depression and anxiety disorders is a tough task due to the similarities in the nature of the symptoms. Vigne et al. have reported that mild OCD and other mental disorders symptoms tend to cluster; however, as severity increases, they tend to differentiate into specific types of disorders [37]. Eddy et al. have revealed that the differential diagnosis of OCD should be based on the identification of obsession-compulsion behaviors because, in OCD, obsession is always accompanied by compulsive rituals that are not observed in other disorders [38]. The World Health Organization (WHO) classified OCD as one of the 10 most debilitating conditions that hinder social growth and quality of life [39].

Post-traumatic Stress Disorder (PTSD)

PTSD is a syndrome that results from exposure to a traumatic event such as threatened death, injury, or sexual assault. PTSD is often associated with functional impairment, suicidal thoughts, and self-injury. Previously, PTSD was classified under anxiety disorders; however, DSM-5 has categorized PTSD under trauma and stress-related disorders [40]. The common symptoms of PTSD include flashbacks of the traumatic event, dissociation, and emotional burden [41]. Sleep problems, hyperreactivity, and avoidance of traumatic event triggers are common manifestations of PTSD. In DSM-5, several changes have been made to the PTSD diagnostic criteria. The updated criteria for exposure to the traumatic event include directly experiencing trauma, witnessing a traumatic event with others, learning of the traumatic event with a close person, and repeated exposure to the details of the adverse events. According to DSM-5, PTSD should be characterized based on the presence of one or more of the following intrusive symptoms after exposure to a traumatic event: recurrent distressing memories; recurrence of dreadful dreams related to the traumatic event; flashbacks; prolonged psychological distress; and marked psychological reactions initiated by internal and external cues that resemble an aspect of the traumatic event. Another key diagnostic measure for PTSD is persistent avoidance of stimuli associated with the traumatic event, including thoughts, feelings, people, places, and conversations. DSM-5 has added new criteria for PTSD, which relate to negative alterations in cognitive processing. These include an inability to remember a key aspect of the traumatic event due to dissociative amnesia, loss of interest in activities, feelings of estrangement, lack of positive emotions, persistent feelings of negativity, mounting blame on oneself, and distorted cognition relating to the traumatic event. The diagnosis of PTSD should be based on the presence of persistent symptoms for over a month [30]. The differential diagnosis of PTSD is very difficult as its symptoms overlap with other anxiety and mood disorders; however, a previous history of a traumatic event can help differentiate it from other disorders [42]. Estimates suggest that almost 3% of the adult population struggles with PTSD at least once during their lifetime. Lifetime prevalence rates for PTSD are estimated to be between 1.9% and 8.8% [43].

Therapeutic approaches

Cognitive Behavioral Therapy (CBT)

CBT is a blend of behavioral and cognitive interventions to reduce maladaptive thinking and ensure positive prospective outcomes. Cognitive interventions are aimed at achieving alterations in beliefs and modification of maladaptive cognitions [44]. Mostly, CBT is a problem-oriented strategy to impart modification in the thought process and cognitive behavior. In most anxiety disorders, this method is considered the first line of treatment. Behavioral and cognitive behavioral therapies have been shown to be time-limited and effective interventions for anxiety disorders [45]. CBT is not a unitary intervention protocol and there are various potential moderators of outcomes that may be clinically important. For SAD, two types of CBT models have been used effectively: the Heimberg model and the Clark and Wells model, both individually as well as in group settings [46]. Using CBT for social anxiety has shown improvement regarding feared social situations by way of cognitive restructuring and in vivo exposure. Typically, the patient is motivated to identify and challenge their beliefs about their social competence and the probability of experiencing negative social evaluation and consequences. The in vivo exposures provide ways to confront feared and avoided social encounters and practice social skills. The effectiveness of CBT in social anxiety has been validated in the meta-analyses by Otte (2011), which confirmed that CBT is by far the most consistently and empirically supported psychotherapeutic option in the treatment of anxiety disorders [47]. Thus, CBT is considered the gold standard in the psychotherapeutic treatment of patients with anxiety disorders.

In a randomized, controlled trial, 488 children between the ages of seven and 17 years, having a primary diagnosis of separation anxiety disorder, GAD, or social phobia were assigned to receive 14 sessions of CBT, sertraline, and a combination of sertraline and CBT, or a placebo drug for 12 weeks. The proportion of children who were rated as very much or much improved on the Clinician Global Impression-Improvement scale for the different approaches was as follows: 80.7% for combination therapy, 59.7% for CBT, and 54.9% for sertraline (p<0.001). Treatment by CBT and sertraline reduced the severity of anxiety in children with anxiety disorders; the combination of the two therapies had a superior response rate [48]. Similar findings have been reported by Jazaieri et al. who investigated the impact of CBT on emotional regulation and satisfaction with life in SAD patients. Their results showed that the CBT group had a higher cognitive reappraisal and satisfaction with life [49]. Bulter et al. examined the individual CBT outcomes in 93 SAD patients in a university outpatient setting. After 20 weeks of CBT, the SAD participants reported a decrease in their social anxiety levels and an improvement in their quality of life. The number of sessions of CBT varied depending on the needs of each individual [50]. Similar findings were shared by Leigh and Clark in their study of teenagers suffering from SAD. They found a significant improvement in SAD levels and quality of life, including improvements in participation in the classroom [51]. Goldin et al. have reported that CBT modulates positive cognitive reappraisal in SAD patients by reducing negative emotions and improving functional connectivity of the brain [52]. Even brief CBT interventions have been demonstrated to be effective in managing SAD symptoms. Pinjarkar et al. showed that brief CBT treatment of only six weeks showed improvement in outcomes in 17 SAD patients [53]. Behera et al. found that CBT along with pharmacotherapy results in better SAD management. Their findings showed that pharmacotherapy alone had inferior outcomes compared to combination therapy with CBT [54].

Different modalities of CBT treatment are currently practiced, such as applied relocation, exposure, and cognitive restructuring. However, upon comparing the different modalities, the results showed that they are equally useful with only a few differences. This shows that all CBT methods in general are good and beneficial. On the other hand, having few to no differences in the clinical results can raise concerns regarding the ineffectiveness of the different modalities of CBT [55]. However, there is growing evidence regarding the effectiveness of recent methods of CBT compared to the older ones, which indicates the necessity of conducting CBT trials among SAD patients [55].

Pharmacotherapy and Its Side Effects

Before initiating drug therapy, the patient should be informed about treatment alternatives, expected outcomes, and potential adverse effects of drugs. Once the treatment is initiated, it should be monitored frequently to assess efficacy. To avoid relapse, the patient should continue taking the medication for at least a year after achieving the desired therapeutic response [56]. The most common classes of drugs used to treat SAD are SSRIs, serotonin-norepinephrine reuptake inhibitors (SNRIs), monoamine oxidase inhibitors (MAOIs), GABA-ergic drugs, benzodiazepines (BZDs), beta-blockers, and other anxiolytics [57]. The first line of treatment for SAD is SSRIs, which work by increasing serotonin levels in the brain by inhibiting serotonin reuptake in neurons and thereby improving communication between neurons [58]. The addition of propranolol to the treatment regimen is controversial. A systematic review and meta-analysis concluded that the routine use of propranolol for SAD is not recommended due to the insufficient number of studies investigating the effects of propranolol on SAD [59].

First Line of Treatment

SSRIs are the first-line agents to treat SAD; they work by increasing the levels of serotonin in the brain by blocking the reuptake of serotonin into neurons. The increase of intra-neuron serotonin improves the transmission of messages between neurons [60]. The FDA has approved several SSRIs including sertraline, paroxetine, and fluvoxamine for SAD. Nausea, dry mouth, headache, insomnia, loose stools, sexual dysfunction, somnolence, sweating, tremor, and weight change are all common SSRI side effects. Additionally, SSRIs may raise the risk of hyponatremia, bleeding, bone fractures, drowsiness, and extrapyramidal symptom [61]. Venlafaxine is an SNRI that can be used to treat SAD as a first-line treatment. Like SSRIs, SNRIs are also very effective; however, while SSRIs raise serotonin levels in the brain, SNRIs raise serotonin and norepinephrine levels. SNRIs can also lead to certain adverse effects, nausea being the most common [62]. Other side effects include suicidal ideation, diarrhea, headache, hypertension, sexual dysfunction, and discontinuation syndrome.

Second Line of Treatment

High-potency BZDs, such as alprazolam and clonazepam, have been shown to be effective as monotherapy in the treatment of SAD symptoms [63]. However, due to abuse risks and ineffectiveness in treating comorbid depression, the Anxiety and Depression Association of America does not recommend BZDs as a first-line therapy for SAD. Gabapentin or pregabalin are used as alternative treatment options for people who have a history of using BZDs or substance use disorder (SUD). Although several randomized controlled trials have shown that phenelzine, a monoamine oxidase inhibitor, is highly effective, its use is restricted due to side effects and dietary restrictions. MAOIs are usually reserved for SAD cases that do not respond to other treatments [64].

## Conclusions

SAD, also known as social phobia, is a chronic debilitating condition marked by the fear of social situations. Social anxiety should be distinguished from normal shyness as the latter is a normal condition whereas the former can have serious repercussions. This phenomenon is prevalent among young adults and it can significantly impact their social growth. SAD can have a devastating impact on the quality of life, negatively affecting relationships, work performance, social engagement, and personal growth, and even leading to substance abuse. The manifestation of anxiety symptoms can range from having issues with attending specific social events to fear of all social interactions based on the severity of the disease, which could be mild, moderate, or severe. LSAS is employed as a common tool to diagnose SAD in individuals. Behavioral therapy and pharmacotherapy are two distinct approaches to the management of SAD symptoms. The combination of both therapies has shown promising outcomes in patients suffering from SAD.
